# A Systematic Review of Randomized Controlled Trials of Telehealth and Digital Technology Use by Community Pharmacists to Improve Public Health

**DOI:** 10.3390/pharmacy8030137

**Published:** 2020-08-04

**Authors:** Philip Crilly, Reem Kayyali

**Affiliations:** Department of Pharmacy, School of Life Sciences, Pharmacy and Chemistry, Kingston University, Kingston upon Thames KT1 2EE, UK; P.Crilly@kingston.ac.uk

**Keywords:** community pharmacy, telehealth, digital health, public health, systematic review

## Abstract

Community pharmacists (CPs) continue to have an important role in improving public health, however, advances in telehealth and digital technology mean that the methods by which they support their customers and patients are changing. The primary aim of this study was to identify which telehealth and digital technology tools are used by CPs for public health purposes and determine if these have a positive impact on public health outcomes. A systematic review was carried out using databases including PubMed and ScienceDirect, covering a time period from April 2005 until April 2020. The search criteria were the following: randomized controlled trials, published in English, investigating the delivery of public health services by community pharmacists using a telehealth or digital tool. Thirteen studies were included out of 719 initially identified. Nine studies detailed the use of telephone prompts or calls, one study detailed the use of a mobile health application, two studies detailed the use of a remote monitoring device, and one study detailed the use of photo-aging software. Public health topics that were addressed included vaccination uptake (n = 2), smoking cessation (n = 1), hypertension management (n = 2), and medication adherence and counseling (n = 8). More studies are needed to demonstrate whether or not the use of novel technology by CPs can improve public health.

## 1. Introduction

At a time when the world is in the midst of a global pandemic [1], community pharmacists need to adapt to a “new normal” in which human-to-human contact between them and their patients/customers can be limited [2]. New approaches to communication and service delivery are needed to keep community pharmacy teams and the public safe from a virus that has no known vaccine and whose long-term impact on society is not yet known [2,3]. Although, currently, the focus for community pharmacy is to deal with the pandemic, there may come a time, in the near future, when the pressures of this subside, and then community pharmacists will need to consider how they continue to deliver public health interventions that deliver positive health outcomes, but in an era of continued social distancing [3]. There are different settings in which pharmacists operate, such as in a community, in a hospital, and in industry; however, for the purposes of this article, the terms pharmacy and pharmacist refer to the community arm of the profession.

In addition to the immediate public health concerns of the coronavirus, other important global health issues still remain, including both communicable (e.g., HIV, tuberculosis, malaria, and hepatitis) and noncommunicable diseases. According to the World Health Organization’s (WHO) 2016 global report on causes of deaths [4], six of the top ten causes of death were from noncommunicable diseases (NCD). NCDs are chronic health conditions, such as heart disease, chronic obstructive pulmonary disease (COPD), and diabetes [5]. Of concern, they are more commonly associated with those of lower socioeconomic status, leading to health inequalities and higher rates of death amongst this group [5]. NCDs have a number of modifiable risk factors including physical inactivity, poor diet and nutrition, smoking, overweight and obesity, alcohol intake, and raised blood pressure. Given that these risk factors are modifiable, and thus can be targeted, much research has been carried out to investigate how their impact on health could be addressed. In fact, the WHO has produced a Global Action Plan for the Prevention and Control of NCDs, with suggested actions to reduce a range of issues including harmful use of alcohol, insufficient physical activity, tobacco use, and unhealthy diets [6]. 

Countries worldwide take a multipronged approach to addressing the abovementioned modifiable risk factors. Strategies employed have included increasing taxes on tobacco, sugar and alcohol, restricting marketing of these products, restricting access to these products through licensing laws, and raising public awareness about the harm that these products can cause [7,8,9,10]. In relation to communicable diseases, sexual health screening and treatment services [11], vaccination administration services [12], and antibiotic stewardship [13] are key strategies to improve public health. 

Those working in the primary health sector, such as community pharmacists, form a key part of the public awareness strategy and, in addition, have an important role to play in treating, screening, and providing brief interventions to those who may be at risk of harm [14]. This aspect of public health has become engrained into the profession of community pharmacy for many years [15]. There is evidence that when pharmacists take a patient-centered approach, incorporating motivational interviewing and health behavior change models, they are able to engage and support the public better in order to make positive lifestyle choices [16]. To date, pharmacists have delivered public health services that have been both effective and cost-effective including alcohol brief interventions [17], weight management [18], smoking cessation [19], sexual health screening and treatment [11], supply of emergency and regular contraception (EHC) [20], diabetes risk assessment [21], influenza vaccination [22], and asthma management [23]. In addition, community pharmacists continue to support their patients by delivering interventions that address medication adherence issues [24] as well as counseling in the correct use of medications [25], in order to improve the health outcomes of the conditions being treated. 

Recent research has highlighted the continued role of pharmacy teams to improve public health and has discussed how digital technology could be used to enhance their role in this domain [15,26]. The use of technology in healthcare has led to the coining of the term telehealth. It is an amalgamation of a number of other terms, such as e-health, mHealth, and mobile health, and describes how health services can be delivered via non face-to-face means [27]. Telehealth tools have been shown to be effective in a number of different settings and for a number of different public health issues [28,29,30,31,32]. Social media in particular has a number of features that could support the public health role of pharmacists. These include the ability to allow the pharmacist to communicate with a patient or customer remotely, a high level of accessibility by all members of the public regardless of demographics, and the ability to improve the health of members of the public who would not normally visit a pharmacy. A number of studies have discussed how technology has already been used to encourage smoking cessation, weight loss, and alcohol reduction [33,34,35]. In a study by Crilly et al. [15], community pharmacists stated that the use of social media and mobile health applications would allow them to have a positive impact on public health. In addition, the public have stated that they would use digital public health tools, if these were delivered by community pharmacy teams [26,36]. Previous research has examined the use of social media platforms for weight loss interventions, in particular [35,37,38]. Such research articles, focusing on the use of digital technology by community pharmacists, are particularly relevant now, given that social distancing is likely to remain in place for some time, meaning that the profession needs to find new ways to continue to deliver public health interventions in spite of the pandemic. 

Working to integrate telehealth and digital health concepts into healthcare interventions introduces challenges related to both health literacy and digital literacy. Health literacy describes, “… the degree to which individuals have the capacity to obtain, process, and understand basic health information and services needed to make appropriate health decisions” [39]. In layman’s terms it describes a member of the public’s ability to interpret the health information that they are given, and then apply it to their own lives while digital literacy, as defined by Gilster in 1997 [40], is, “…the ability to understand and to use information from a variety of digital sources… [it is] literacy in the digital age.” Of interest, a 2016 study [41] highlighted that those with low health literacy were also less likely to use digital health tools or to find them easy to use, indicating that these concepts can be linked, and can even increase the incidence of health inequalities. As a result, it is important that healthcare professionals, including pharmacists, not only consider the health literacy but also the digital literacy of their patients and customers. In addition, it is important that healthcare professionals develop their own digital literacy skills so that they can support their patients and customers to reap the benefits of technological advances to improve their health. A 2016 systematic review raised concerns about regarding pharmacies supporting the digital training of their workforce [42]. This review highlighted that while a pharmacy is using technology on a daily basis it does not appear to have adapted a structured training approach or development standards for its workforce. This raises concerns, that is, while the public are embracing novel technology and turning to it for health and wellbeing advice, the use of technology by pharmacists and its impact on the health of those who interact with pharmacists is still unknown [27]. There is risk that the profession could being left behind, and would no longer be deemed to be the most accessible healthcare professional, as we are now.

Therefore, we conducted a systematic review of the literature to understand how pharmacists have used telehealth and digital technologies to improve public health related outcomes, addressing NCDs as well as communicable diseases and medication-related topics. 

## 2. Materials and Methods 

### 2.1. Design of the Study 

This systematic review was carried out following the recommendations of the Preferred Reporting Items for Systematic Reviews and Meta-Analyses (PRISMA) Statement [43].

The review was carried out between March 2020 and May 2020 to identify any papers published between April 2005 and the end of April 2020. 

### 2.2. Data Sources and Search Terms 

PubMed, Medline, Science Direct, Web of Science, and Scopus were searched for articles that reported outcomes associated with the use of telecommunication or digital communication technologies by pharmacists for the purposes of improving public health. Medical Subject Heading (MeSH) terms and keywords (KW) were identified and a search strategy was developed using the PICO (Population, Intervention, Comparator, Outcome) framework. MeSH terms and KWs for community pharmacy included pharmacy (MESH), pharmacies (MeSH), community pharmacy services (MeSH), and retail pharmacy (KW). MeSH terms and KWs for telecommunicaitons and digital communication technology included telephone (MeSH), social media (MeSH), mobile applications (MeSH), internet (MeSH), Facebook (KW), Twitter (KW), Instagram (KW), and YouTube (KW). MeSH terms and KWs for public health interventions and outcomes included public health (MeSH), smoking cessation (MeSH), weight-loss (MeSH), health (MeSH), sexual health (MeSH), and alcohol brief intervention (KW). The full PICO search criteria used on PubMed is available in Supplementary Materials. Paper references were used to find additional studies, as was Google Scholar and the authors’ institutes own library search engine. 

### 2.3. Study Selection and Definitions

For this systematic review, we included the following studies that were: (1) randomized controlled trials (RCTs); (2) full articles published in peer-reviewed journals; (3) included a telehealth or digital technology element used for either interaction between a community pharmacist and customer/patient, such as telephone, email, online discussion boards, social media, smartphone mobile application, or for patient use alone as part of a community pharmacy intervention; and (4) reported public health interventions and outcomes. 

### 2.4. Data Extraction

The authors (P.C. and R.K.) identified studies’ titles that suggested that they met the inclusion criteria of the review. Further to the initial screening, abstracts and full articles were reviewed and removed if the primary objectives of the paper did not investigate the use of telehealth and digital technologies by community pharmacists for public health purposes and were not run as RCTs. A template was created in Excel, which both authors used to extract data including intervention variables (location of intervention and recruitment criteria, topic of delivered public health intervention, type of telecommunication or digital communication technology used, and duration of the intervention), participant variables (mean age, gender, and ethnicity), outcomes variables (e.g., quit smoking rates, reduction in weight, reduction in alcohol consumption, and treatment of a sexual transmitted disease). Face-to-face meetings were conducted between the authors to compare each other’s identified studies. 

### 2.5. Methodological Quality of RCTs and Risk of Study Bias

The Jadad scale [44], which gives each study a score out of a total of 5 based on the degree of randomization, blinding, and withdrawal/drop-out was used to assess the methodological quality of the reviewed RCTs. A study with a low risk of methodological bias was given a score of 4 or more, while a study with a high risk of methodological bias was given a score of 0 or 1. A moderate risk of methodological bias prompted a score of 2 or 3. In addition, Cochrane’s Risk of Bias (RoB 2) tool was also used to assess the risk of bias in the reviewed randomized trials [45]. The RoB 2 tool gives an overall risk of bias related to bias in the following five domains: randomization, deviations from intended interventions, missing outcome data, measurement of the outcome, and selection of the reported result. RCTs are graded as low risk, some concerns, or high risk based on the combined grading of the five different domains. Ethical approval was not required for this study. 

## 3. Results

**Figure 1:**
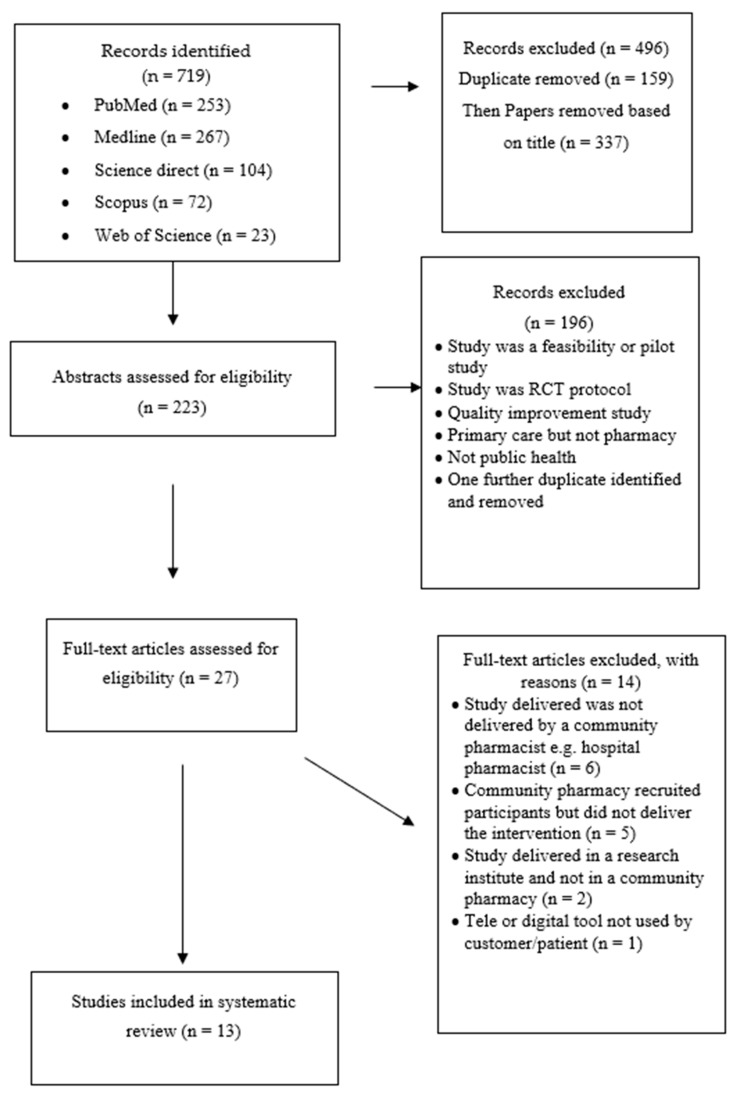
Flow diagram of the study selection process.

Using the criteria outlined, the initial search identified 719 potential papers. Duplicates were removed, and then papers were quickly screened by title. Articles not meeting the eligibility criteria were removed. This left 223 potential papers. Articles were, then, screened by abstract for inclusion criteria, and then the remaining articles were screened as full articles for inclusion. In total, 13 studies were included in this review. The search process is outlined in Figure 1 and the studies used as part of the review are summarized in Table 1. The studies were conducted around the world, including the USA (n = 7), [46,47,48,49,50,51,52] the UK (n = 1) [53], Australia (n = 1) [54], the Netherlands (n = 3) [55,56,57], and Canada (n = 1) [58]. Public health topics addressed in the studies included vaccination rates [46,47], smoking cessation [54], medication adherence [48,49,52,53,55,57,58], medication counseling [56], and hypertension management [50,51]. The majority of the studies used telephones as their intervention tool [46,47,48,49,52,53,56,57,58] while only one study used an mHealth app [55], two studies used a tele-monitoring device [50,51] and one study used photo-aging software [54]. Despite smartphones now including video conferencing features, none of the reviewed studies employed this technology.

### 3.1. Public Health Topics

The main types of teledigital health interventions and outcomes found in this review were medication adherence and counseling, hypertension management, vaccination uptake, and smoking cessation. Most of the reviewed studies showed a positive impact of the use of telehealth and digital health tools, such as telephones and mobile health applications, on participants’ health outcomes.

#### 3.1.1. Vaccination Uptake

Two studies [46,47], both based in the USA, investigated the use of interventions to increase vaccination uptake rates and both used automated telephonic prompts versus usual care. One of the studies showed a significant increase in the uptake of the herpes zoster vaccination in the intervention group, whereas the other study, looking at pneumococcal and herpes zoster vaccination uptake, did not see differences between the intervention and control groups. Of note, in the study where no differences in uptake were found, only 28.2% of participants listened fully to the first telephonic prompt. Those who did listen to the full call were more likely to have a vaccination administered.

#### 3.1.2. Smoking Cessation

One study, based in Australia, investigated how photo-aging software could be used by community pharmacists to promote smoking cessation [54]. Participants were shown images of how they would look as an older person if they were to continue smoking or to quit. There was a significantly higher quit rate at 6 months among those who had used the photo-aging software vs. the usual care group. In addition, those in the intervention group showed lower levels of dependence on nicotine by the end of the study as measured by Fagerström score (*p* < 0.001).

#### 3.1.3. Medication Adherence

Seven studies investigated the impact of telehealth or digital interventions by community pharmacists to improve medication adherence [48,49,52,53,55,57,58]. One was based in the UK, three in the USA, two in the Netherlands, and one in Canada. The UK, USA, Canadian, and one of the Netherlands [57] studies used telephone calls as the intervention tool while the other Netherlands study [55] used an mHealth app. The UK study investigated the UK advanced pharmacy service, the New Medicine Service (NMS), a service in which patients with certain chronic health conditions are supported when newly prescribed medications as a means to improve adherence [59]. An initial feasibility study had indicated that a telephone-based pharmacy advisory service had a significant impact in reducing non-adherence to medication [60], however, the follow-up study suggested that this difference was lost after 10 weeks [53]. 

The USA studies investigated patient adherence to antidepressants [49] and medication for chronic health conditions [49,52]. One of the USA studies [49] showed a significant improvement in patient adherence to medication for type 2 diabetes whilst the antidepressant study showed an increase in patient knowledge and medication beliefs, but not an improvement in adherence. 

The Canadian study looked specifically at antibiotic counseling with patients in the intervention group being telephoned by the pharmacist on day three of their antibiotic therapy [58]. The telephone intervention was deemed to have been effective in identifying and managing drug related problems but did not have an impact on adherence or severity of infection symptoms versus the usual care group.

The earlier of the Netherlands’ studies [57] investigated medication adherence to antidepressants, bisphosphonates, statins, or Renin-Angiotensin-System (RAS) inhibitors in patients taking these medications for the first time. The study demonstrated that adherence to RAS inhibitors was improved through telephone counseling, however, this effect was not noted for antidepressant therapy. The later Netherland’s study used an mHealth app to improve adherence in adolescent asthmatics [55]. The study showed that the mHealth app had a statistically positive effect on medication adherence (*p* = 0.04) but did not affect patient quality of life or asthma control [55]. 

#### 3.1.4. Medication Counseling

The medication counseling study was carried out in the Netherlands [56] and was a pre-cursor to a medication adherence study described above [57]. While the study was ultimately interested in medication adherence, adherence was not one of the measured outcomes. Instead, the authors felt that it was more important to, “…assess the impact on the pathway that ultimately leads to adherent behavior” [56] and so they assessed the effect of telephone counseling on general satisfaction with counseling, satisfaction with information and beliefs about medicines. The majority of patients in the study stated that the telephone call had added value, however, there were no differences in patient satisfaction with the information provided versus usual care. 

#### 3.1.5. Hypertension Management

Two studies (initial and follow-up), based in the USA, investigated the use of home blood pressure telemonitoring with community pharmacist management [50,51]. Patients measured their BP on a monitor that then transmitted this reading to the pharmacist. The pharmacist then called the patient to discuss medication adherence as well as lifestyle and treatment issues. There were significant improvements in BP control for the intervention group at 6 months (71.8% controlled, *(p* < 0.001)), 12 months (71.2% controlled, (*p* = 0.005)), and 18 months (71.8% controlled, (*p* = 0.003)) and the intervention was noted to have sustained effects for at least 24 months. The intervention group sustained significantly lower SBP and DBP versus control group for 24 months from baseline.

### 3.2. Methodological Quality of RCTs and Risk of Study Bias

The Jadad tool (see Supplementary) indicated that while all of the reviewed studies were randomized, and included details about the method of randomization, only one study [53] was double-blinded. The same study was also the only one to describe withdrawals/dropouts. The median score was two, indicating a moderate risk of methodological bias for the studies in the review. In fact, all but two of the studies reviewed were deemed to have a moderate risk of methodological bias [46,47,48,49,50,51,52,54,55,56,58]. The remaining studies were deemed to have a low risk of methodological bias [53,57]. The Cochrane RoB-2 tool highlighted that two of the reviewed studies had a low risk of bias [53,57], while the remaining 11 studies all had “some concerns” in relation to their risk of bias [46,47,48,49,50,51,52,54,55,56,58] (see Table 1). Missing outcome data and measurement of the outcome were the domains that raised the risk of bias in the 11 studies [46,47,48,49,50,51,52,54,55,56,58].

## 4. Discussion

This systematic review of RCTs evaluating the use of telehealth and digital technology by community pharmacists to improve public health has identified a number of studies that demonstrated the effective use of these tools. 

The telephone was found to be the most commonly used intervention tool. Of note, no studies used social media as an intervention tool. These findings match those of a previous review which highlighted that there was very little evidence of measurable health outcomes following the use of social media, in particular, by community pharmacists to improve public health [34]. 

The use of telehealth and digital technology by community pharmacists is not a new research area [15,26,34,61,62], however, there are very few RCTs showing demonstrable impacts on participants’ health outcomes. Rather, research appears to be anecdotal detailing pharmacists and patients’ perceptions and opinions about their use of these tools [15,26,34,61,63]. By bringing together all of the RCTs, it is possible to identify successes to date and discuss how telehealth and digital technology could be utilized more effectively in the future. 

The lack of use of video conferencing technology is interesting; however, one study that did not meet the inclusion criteria for this review did show promise in the use of video conferencing software [64]. Video conferencing was used to support those on multiple medications by offering an opportunity to identify medication-related problems (MRP) and provide patient education. Patient satisfaction with the technology was noted to be high and pharmacists found the technology straightforward to use [64]. Benefits that could be achieved with video conferencing include face-to-face discussions to allow the pharmacist to view patient/customer body language as a way of determining health, an opportunity for the pharmacist to demonstrate particular behaviors to educate patients/customers, and as a way to build a trusting relationship between the pharmacist and the patient/customer.

Another point highlighted in this review was the limited use of “novel” technology by community pharmacists. A mobile health application was only used in one study [55], whereas social media was not used in any study. Factors relating to the digital literacy of the pharmacy workforce could be a key issue holding back the use of these tools [42]. A number of studies have highlighted that while many within the pharmacy workforce had high levels of digital literacy, most did not use technology in their work life [65]. Those who used these tools in their personal life were more likely to use them in their work life, but factors relating to concerns about confidentiality and privacy prevented them from using them more [15]. 

The public health topics for which pharmacists used telehealth or digital technology in studies in this review were limited in scope. Many topics focused on medication counseling and adherence which are more traditional roles of the community pharmacist [48,49,52,53,55,56,57,58]. There were only five studies that addressed public health topics with the main outcomes being unrelated to medication adherence or counseling [46,47,50,51,54]. The other topics addressed were vaccination uptake, smoking cessation, and hypertension management. Given that community pharmacists deliver public health interventions including weight management, sexual health, and alcohol use, among others, it is surprising that no studies addressed these important and relevant public health challenges. A study by Crilly et al. [26] pointed out that if the public were to use social media and mobile health apps for public health reasons, then topics such as weight management would be at the top of their list. By community pharmacy not embracing these telehealth and digital mediums they could be missing opportunities to support the public to meet a need that they have. 

This review has highlighted the positive role that telehealth and digital technology can play in supporting community pharmacists to deliver public health services. As such, these tools need to continue to be integrated within community pharmacy services. These tools will be particularly useful for those patients and customers who are not able to visit community pharmacies in person. Given that the world is currently in the grips of a global pandemic, it is clear that offering community pharmacy users’ methods of communication other than only face-to-face would allow the profession to continue to have an impact on public health regardless of the ability to physically meet [2,3]. The use of the telephone, in particular, has consistently shown to have a positive impact on participant health outcomes [46,47,48,49,52,53,56,57,58]. 

The use of more novel telehealth and digital technology such as mobile health applications, social media, and video conferencing would benefit from further research. Anecdotal evidence exists suggesting that community pharmacists do use these tools for public health purposes; however, future studies need to address how these compare to usual care and telephone only interactions [15,34,61].

This study has demonstrated that if community pharmacy is to progress beyond the traditional face-to-face method of communication, then, more studies are needed that address the use of novel technology for a variety of public health topics. Those who commission public health services should be consulted to determine whether a framework for delivering community pharmacy public health services is needed. A blueprint could be created that community pharmacists could use to develop new services. This blueprint would detail how to engage particular demographics, which types of technology are best suited for addressing particular health topics and whether combining technology with face-to-face interaction is needed. 

Public awareness of the role of community pharmacy in public health also needs to be raised, as a recent study noted that the public would visit their GP or use an online resource to look for health advice rather than consulting a pharmacist [26]. As such, community pharmacy needs to incorporate the methods of communication and the new types of technology that the public are using in order to be able to engage them in key public health issues. 

### Limitations

There were a number of limitations associated with this systematic review. Firstly, this review was interested only in RCTs. As a result, some other interesting studies that used alternative methods may have been missed. Secondly, each of the studies identified had different primary outcomes, therefore, direct comparison of the results was not possible. Finally, due to the everchanging nature of telehealth and digital technology, some novel tools used by community pharmacists for public health purposes may have been missed. 

## 5. Conclusions

This systematic review highlights how telehealth and digital technology is being used by community pharmacists to improve public health. We found that telephone calls and automated telephonic prompts were the most commonly used alternative method of communication to face-to-face discussions. There were limited studies on the use of more novel technology, with only one study using a mobile health app, one study using a remote health monitoring device, and no studies using social media. In addition, medication adherence was the main public health topic addressed in the studies, with other public health issues such as overweight and obesity, mental health, and sexual health not being investigated at all. 

Studies that evaluate the use of more novel technology and a broader range of public health topics are needed to understand if community pharmacists can enhance their role in these domains and meet the needs of a digitally literate customer base. 

## Figures and Tables

**Table 1:** Summary of study characteristics.

**Study Author and Year Research Completed**	**Intervention Design and Study Population (I = Intervention and C = Control)**						**Primary Outcome**	**Results**	**Risk of Bias (RoB-2)**
	**Study Location**	**Duration**	**Tele or Digital Medium Used**	**N**	**Mean Age (Years)**	**% Female**			
Vaccination Uptake									
Hess (2013) [47]	USA	14-months	Automated telephonic prompt	I = 5599 C = 6383	I = 72.9 C = 71.8	Not stated	The number of HZ vaccines administererd to study participants (adults 60 years and over) during the time period of March to May 2007	In the intervention group, 146 vaccinations were administered to the study participants. In the control group, 46 vaccinations were administered. This accounted for HZ vaccination rates of 2.6% vs 0.72% for intervention and control groups, respectively, representing a significant improvement in vaccination rates.	Some concerns
	Participants: Adults over 60 years who were not vaccinated for herpes zoster (HZ) Intervention: Participants received a 30-s automated telephone prompt advertising that they should speak to their pharamcist about their risks of HZ and the availability of the vaccine. Control: Did not receive a telephone prompt								
Stolpe and Choudhry (2019) [46]	USA	10-months	Automated telephonic prompt	I = 11,009 C = 10,962	I = 63.2 C = 63.3	I = 56.9 C = 57.7	The proportion of study participants who were administered at least one of their missed vaccines between the time period of March 2015 and January 2016.	In the intervention group, 236 vaccinations were administered to the study participants. In the control group, 225 vaccinations were administered. This accounted for vaccination rates of 2.14% and 2.05% for intervention and control groups respecitvely. This was not a significant differrence. Only 33.3% of intervention participants listened to the full telephone call. Of those who did, they were more likely to get vaccinated than controls.	Some concerns
	Participants: Adults aged over 19 years old who were scheduled to have an automated telephone call from their community pharmay, e.g., to remind them to refill their medication Intervention: Participants received an additional prompt within the call and were offered either pneumococcal or HZ vaccine or both, depending on their vaccination record. Three call attempts were made in total. Control: Received their scheduled automated telephone call but without the additional vaccination offer.								
**Study Author and Year Research Completed**	**Intervention Design and Study Population (I = Intervention and C = Control)**						**Primary Outcome**	**Results**	**Risk of Bias (RoB-2)**
	**Study Location**	**Duration**	**Tele or Digital Medium Used**	**N**	**Mean Age (Years)**	**% Female**			
Smoking Cessation									
Burford et al. (2013) [54]	Australia	12-months	Internet-based photo-aging software	I = 80 C = 80	I = 24.2 C = 25.1	I = 55 C = 45	The number of successful smoking quit attempts, confirmed by carbon monoxide (CO) breath test, measured at 1, 3 and 6 month follow-ups. In addition, nicotine dependence was measured via the Fagerström scale	11 (13.8%) of intervention group were non-smokers at 6 months vs 1 (1.3%) of control group (*p* = 0.003). This was significant and confirmed by CO monitor. Change in Fagerström score from baseline at 6 months was −0.26 for control group and −1.88 for intervention group. 14% of the control group moved to a lower category of the Fagerström score versus 51% of the intervention group (*p* < 0.001)	Some concerns
	Participants: Adults aged 18–30 years old, who were smokers, without facial hair, who did not suffer from body dysmorphia and who did not take nicotine replacement therapy or medication for nicotine dependence. Intervention: Participants received 2 min of smoking cessation advice from a pharmacist. In addition, Face aging software, APRIL, was used to create aged images of faces from a digital photograph. In addition to the using the normal wrinkling algorithm, the images were also adjusted to compare how participants would age as a smoker versus as a non-smoker. Control: Participants received 2 min of smoking cessation advice from a pharmacist								
Medication Adherence									
Rickles et al. (2005) [48]	USA	12-months	Telephone call	I = 31 C = 32	I = 37.8 C = 37.5	I = 80.6 C = 87.5	The number of times study patients spoke to their pharmacist about their new medication (whether during intervention call or when visiting the pharmacy). In addition, changes in patient knowledge, beliefs, adherence, and depression symptoms at 3 and 6 months.	The intervention had a significant and positive effect on the number of times patients spoke to their pharmacist about their new medication (*p* < 0.001). In addition, patient knowledge, medication beliefs, and perceptions of progress were significantly better in the intervention group (*p* < 0.05). No significant difference was noted in adherence, however, fewer doses were missed in the intervention group. No significant difference was noted in depressive symptoms.	Some concerns
	Participants: Adults aged over 18 years old on a newly prescribed antidepressant (within the previous 4 months before recruitment). Intervention: Participants were telephoned by their pharmacist and taken through Pharmacist-Guided Education and Monitoring (PGEM). This involved three phone calls at monthly intervals, the first lasted 20 min and assessed medication related issues and education. The second and third calls lasted 10 min each and checked progress with medication and helped with problem management. Control: Usual care that pharmacists typically provide for patients on new medications.								
**Study Author and Year Research Completed**	**Intervention Design and Study Population (I = Intervention and C = Control)**						**Primary Outcome**	**Results**	**Risk of Bias (RoB-2)**
	**Study Location**	**Duration**	**Tele or Digital Medium Used**	**N**	**Mean Age (Years)**	**% Female**			
Medication Adherence continued…									
Beaucage et al. (2006) [58]	Canada	2-months	Telephone call	I = 126 C = 129	I = 47 C = 49	I = 55 C = 60	The number of drug-related problems participants reported. In addition, the number and severity of infection symptoms following start of antibiotic treatment. Patient adherence to treatment, as well as patient satisfaction.	53% of intervention participants reported drug related problems versus only 8% in the control arm (*p* < 0.001). These were mostly noted on the phone call and included adverse drug reactions and drug interactions. Differences in the number of infectious symptoms and severity of symptoms between control and intervention group were small and not significant. Adherence to treatment and satisfaction did not differ across the groups.	Some concerns
	Participants: Adult patients with a new prescription for oral antibiotic treatment, whose treatment would last between 5 and 14 days, and who had access to a telephone. Participants had a private consultation with the pharmacist about their antibiotic, potential side effects, and adherence to treatment. Intervention: Referred to the pharmacist telephone follow-up intervention (PTFI). Patient received a phone call from their pharmacist on day 3 of antibiotic treatment to discuss treatment, side effects, adherence and any questions they had. Control: Usual care. Afer intial private consultation patients were invited to contact pharmacist, if needed.								
Nietert et al. (2009) [52]	USA	7 months	Telephone call	PI = 1018 FI = 1016 C = 1014	PI = 59.9 FI = 60.6 C = 59.7	Not stated	The primary outcome was the number of days from the date that a patient was declared at least 7 days overdue to the date of the next prescription refill.	There were no significant differences in primary outcomes by treatment arm. Pharmacists contacted 81% of those patients in the phone patient intervention. Of those, 19.2% stated that they were waiting to switch to another medication and 4.1% stated that they were planning to stop the medication.	Some concerns
	Participants: Patients who were at least 7 days overdue for a medication used to treat a chronic disease (diabetes, hypertension, hyperlipidaemia, heart failure, depression, and psychosis). Usual care: Prescriptions were filled when requested by patients. Phone patient (P) intervention: Pharmacist reminded patient that they were overdue picking up medications, asked why they were overdue, reminded the patient of the importance of taking medications regularly and helped the patient to overcome barriers. Fax physician (F) intervention: Pharmacist faxed prescriber with information about patients overdue collecting medication and asked them to return patient disposition codes via fax to the pharmacy.								
**Study Author and Year Research Completed**	**Intervention Design and Study Population (I = Intervention and C = Control)**						**Primary Outcome**	**Results**	**Risk of Bias (RoB-2)**
	**Study Location**	**Duration**	**Tele or Digital Medium Used**	**N**	**Mean Age (Years)**	**% Female**			
Medication Adherence continued…									
Odegard and Christensen (2012) [49]	USA	18 months	Telephone call	I = 120 C = 145	I = 65 C = 61	I = 53.8 C = 50.3	Determination of medication adherence to type 2 diabetes medication (days late at refill, percentage with a refill gap at 6 days, medication possession ratio (MPR) at 6 and 12 months	Significant improvement in medication adherence in intervention group, based on MPR ratio. The impact was particularly noticed in those participants who had a baseline MPR of 0.80 or less. MPR significantly improved in intervention group at 12 months (*p* = 0.001)	Some concerns
	Participants: Adults over 18-years old on at least one oral prescription diabetes medication who were late for refills. Intervention: Participants were contacted by their pharmacist by telephone and guided through the 4 A’s (ask, advise, assist and arrange), a model used successfully for changing behaviour in smoking cessation. The pharmacist asked if the patient’s medication had run out, why they had been late to order their medication, if they were having any challenges with their medication, and to discuss a self-management plan. A follow-up call occurred between 1 week and 1 month later. Control: Pharmacist discussed ahderence and ordering medication on time at in-person medication refill collections.								
Kooij et al. (2016) [57]	Netherlands	30 months	Telephone call	I = 3094 C = 3627	I = 56.9 C = 59.0	I = 57.7 C = 54.6	The primary outcome was patients’ refill adherence measured using the Medication Possession Ratio modified (MPRm) in the year following start of medication therapy. MPRm > 80% considered adherent.	3094 patients were in the intervention arm, however, only 1054 (34%) received a telephone call. Reasons why patients did not receive a telephone call included: no telephone number available, patient not interested, and patient could not be reached. The analysis of the primary outcome was based on those in the intervention arm who received the phone call. The mean MPRm was 74.7% in intervention arm and 74.5% in the control arm. The proportion of patients who had an MPRm >80% was 69.0% in the intevention arm and 69.9% in the control arm. Patients taking RAS inhibitors were more likely to be adherent in the intervention arm compared to the control arm. The intervention had no benefit to adherence to patients on antidepressants.	Low risk
	Participants: Adults over 18-years old, who speak Dutch or same language as pharmacist and taking an antidepressant, a bisphosphonate, a statin, or a Renin-Angiotensin-System (RAS) inhibitor for the first time. Usual care: Patients receive both or and written information about medication from a pharmacy technician. Patients are given two weeks of new medication and at first refill are asked about their experience. Further counseling given if needed. Intervention: Usual care plus pharmacist telephoned patient between 7 and 21 days after first prescription. The call addressed need for information, medication intake behaviour, barriers including side effects and concerns or beliefs about the medication.								
**Study Author and Year Research Completed**	**Intervention Design and Study Population (I = Intervention and C = Control)**						**Primary Outcome**	**Results**	**Risk of Bias (RoB-2)**
	**Study Location**	**Duration**	**Tele or Digital Medium Used**	**N**	**Mean Age (Years)**	**% Female**			
Medication Adherence continued…									
Kosse et al. (2019) [55]	Netherlands	10 months	mHealth app	I = 87 C = 147	I = 15.0 C = 15.2	I = 55.2 C = 51.0	To deteremine patient self-reported adherence to asthma medication, measured with the Medicine Adherence Report Scales (MARS) tool. Higher scores indicate higher adherence. Secondary outcomes asthma control and quality of life	Adherence of patients who previously had poor adherence increased in intervention group; adherence rates in control group decreased (*p* = 0.04). No significant difference observed between intervention and control groups on asthma control (*p* > 0.05) or quality of life (*p* > 0.05).	Some concerns
	Participants: Asthma patients aged between 12–18 years old, who own a smartphone and have filled at least two prescriptions for inhaled corticosteroids or combination steroid with bronchodilator inhaler in the previous 12 months Intervention: Usual care (as described in the control) plus 6 months access to the ADAPT intervention. ADAPT is a smartphone application connected to software in the patients community pharmacy. The application targeted non-adherent behaviours. The pharmacist could control its settings, review patient use of the application and chat with the patient. Control: Usual care, meaning that patients received instruction on how to use inhaler at first dispensing, and automated system to detect underuse of inhaled corticosteroid or overuse of bronchodilator.								
Elliott et al. (2020) [53]	UK	14 months	Telephone call	I = 251 C = 253	I = 59.5 C = 59.3	I = 49.8 C = 53.4	To determine the self-reported adherence or non-adherence to medication at 10 weeks and 26 weeks follow-up. Adherence was assessed by telephone and was defined as missing medication without agreement with a medical professional in the previous 7 days.	133 (70.7%) out of the 188 who could be contacted at 10 weeks were adherent in the intervention arm versus 60.5% (115/190 who could be contacted) in the control arm (*p* = 0.037)—significant difference. At week 26, 65.6% in the intervention group were adherentas compared with the 57.1% in the control group (*p* = 0.113)—no significant difference. Therefore, statistical difference in adherence between groups was lost after week 10.	Low risk
	Participants: Patients aged 14 years old and over who phsyically present in the pharmacy with a prescription for a new medicine for a predefined long-term medical condition. Intervention: The intervention comprises of a two parts that can be carried out either face-to-face or over the telephone. The first “intervention” happens 7–14 days after the first one-to-one consulation. The “follow-up” then happens 14–21 days after that. The pharmacist will ask about adherence at these meetings. The whole process should be covered within a maximum of 5 weeks. Control: The pharmacists usual advice when a patient presents with a prescription for a new medicine. No follow-up was offered.								
**Study Author and Year Research Completed**	**Intervention Design and Study Population (I = Intervention and C = Control)**						**Primary Outcome**	**Results**	**Risk of Bias (RoB-2)**
	**Study Location**	**Duration**	**Tele or Digital Medium Used**	**N**	**Mean Age (Years)**	**% Female**			
Medication Counseling									
Kooy et al. (2015) [56]	Netherlands	16 months	Telephone call	I = 94 C = 117	I = 59.9 C = 62.2	I = 52 C = 62	1. Patient satisfaction with pharmacist counseling (using the Consumer Quality Index (CQI) plus additional quesitons for intervention arm); 2. Satisfaction with information provided (using Satisfaction with Information about Medicine Scale (SIMS); 3. Beliefs about medicine.	Only 56 of the 94 patients in the intervention arm actually received telephone counseling. Some reasons for not providing counseling included: patient could not be contacted and patient refusal. Usual care participants’ satisfaction with counseling was 31% versus 63% in intervention group who received counseling. Men were more likely to prefer telephone counseling than women (*p* < 0.05). 74% said telephone counseling had an added value.	Some concerns
	Particiapnts: Adults aged 18 years or older who filled a first time prescription for an antidepressant, a bisphosphonate, an antilipaemic, or a Renin-Angiotensin-System (RAS) inhibitor. Intervention: Usual care (as defined below) plus telephone counseling by a pharmacist. Telephone call covered actual intake of medication, barriers to medication use, and information needs about medication. Pharmacist used the Health Belief Model (HBM) to direct the counseling. Usual care: Dutch guidelines on counselling for a first prescription of a new medication. Covers an exploration of the patient’s needs and experiences with medication.								
**Study Author and Year Research Completed**	**Intervention Design and Study Population (I = Intervention and C = Control)**						**Primary Outcome**	**Results**	**Risk of Bias (RoB-2)**
	**Study Location**	**Duration**	**Tele or Digital Medium Used**	**N**	**Mean Age (Years)**	**% Female**			
Hypertension Management									
Margolis et al. (2013) [51]	USA	25 months;	Blood pressure telemonitor	I = 228 C = 222	I = 62.0 C = 60.2	I = 45.2 C = 44.1	The number of participants who had controlled BP at 6, 12, and 18 months. Change in SBP and DBP were also monitored, as were patient satisfaction with care.	At 6 months, BP was controlled in 71.8% of intervention group versus 45.2% of control arm (*p* < 0.001). At 12 months BP was controlled in 71.2% of intervention group versus 52.8% of control arm (*p* = 0.005). At 18 months, BP was controlled in 71.8% of intervention arm versus 57.1% in control arm (*p* = 0.003). SBP was significantly lower in the intervention group versus control group at 6, 12, and 18 months.	Some concerns
	Participants: Adult patients with elevated blood pressure (BP) (systolic BP > 140 mmHg or diastolic BP > 90 mmHg) at their two most recent primary care visits. Patients had to have uncontrolled BP based on 3 BP measurements. Intervention: Patients receievd a home BP monitor that stored and transmitted their readings to a website accessible by pharmacist. Pharmacists met them for 1 h initially to discuss BP management and goal setting. Patients submitted at least 6 weekly BP measurements. During the first 6 months, pharmacists and patients had telephone calls every two weeks until BP controlled for 6 weeks, then calls became monthly. From months 7–12 the phone calls were every two months. After 12 months, the BP monitor was returned. Pharmacist telephone calls discussed lifestyle, medication adherence, and goal setting. Treatment intensification recommended in some instances. Control: Management of BP by a physician and referral to pharmacist for medication therapy management when needed.								
Margolis et al. (2018) [50]	USA	81 months	Blood pressure telemonitor	I = 162 C = 164	I = 62.0 C = 60.0	I = 45.1 C = 42.7	Changes in SMP and DBP from baseline to 54 months	Mean SBP in intervention group reduced by 2.5 mmHg versus 1.0 mmHg in the control group. The intervention group sustained significanttly lower SBP and DBP versus control group for 24 months from baseline.	Some concerns
	A follow-up study to the research described above by Margolis et al. [52] in 2013.								

## Supplementary Materials

The following are available online at https://www.mdpi.com/2226-4787/8/3/137/s1, Table S1: PubMed search terms used, Table S2: Jadad scoring of reviewed articles.
